# Chitosan as an Elicitor in Plant Tissue Cultures: Methodological Challenges

**DOI:** 10.3390/molecules30173476

**Published:** 2025-08-24

**Authors:** Moumita Roy Chowdhury, Mizgin Mehmet, Jit Mukherjee, Anirban Jyoti Debnath, Katarína Ražná

**Affiliations:** 1 Faculty of Agrobiology and Food Resources, Institute of Plant and Environmental Sciences, Slovak University of Agriculture in Nitra, Nitra 94976, Slovakia; 2 Faculty of Agriculture, Department of Field Crops, Ege University, Izmir 35100, Türkiye; mehmetmizgin36@gmail.com; 3 Department of Computer Science and Engineering, Birla Institute of Technology, Mesra 835215, India; jit.mukherjee@bitmesra.ac.in; 4 52/6 Parui Paka Road, Near-Bagbari, Kolkata 700061, India; anirbandebnath@ymail.com

**Keywords:** chitosan (CTS), elicitation, plant tissue cultures, methodology

## Abstract

Chitosan (CTS) is a biodegradable and biocompatible biopolymer derived from chitin. Thanks to its diverse biological activities and environmentally friendly nature, it has emerged as a promising agent in plant tissue culture. Recent studies have highlighted its role as a natural elicitor that can enhance plant growth, seed germination, and the biosynthesis of secondary metabolites in vitro. In plant tissue culture, it acts as a biotic elicitor, mimicking a pathogen attack and activating the pathogenesis-related proteins to induce secondary metabolite production. In vitro tissue culture is a scientifically meaningful and cost-effective approach to testing the elicitation mechanisms of various abiotic elicitors, including CTS. However, the methodology of CTS elicitation in plant tissue cultures is not straightforward or uniform due to the differences in the CTS origin, molecular weight, and degree of deacetylation, all of which directly affect solubility. This review summarizes the methodological approaches to the use of CTS in plant tissue culture elicitation and highlights specific features of these procedures.

## 1. Introduction

Being sessile in nature, plants face a wide range of biotic and abiotic stresses on a daily basis. Biotic stress encompasses factors such as pathogens, parasitic organisms, and herbivores, while abiotic stress includes submergence, salinity, extreme temperatures, drought, heavy metal (HM) exposure, and nutrient deficiencies [1,2]. These stresses hinder plant growth and development, compromise genome stability, and, eventually, reduce crop yield and productivity [3,4,5]. To withstand these stresses, plants have developed various multi-level and complex response mechanisms involving stress sensing, transcriptional and translational processes, post-transcriptional modifications, epigenetic modifications, signaling pathways, and different metabolic processes, including the production of plant secondary metabolites (PSMs) [6,7,8]. To mitigate these adverse effects of stress on crops, productive strategies have been developed, including the development of stress-tolerant or stress-resistant cultivars, the use of plant growth regulators and osmoprotectants, and the use and management of nutrients, arbuscular mycorrhiza inoculation, seed priming, and grafting [9]. Among these strategies, elicitation, an approach involving the enhanced synthesis of PSMs, is gaining traction due to its role in the protection against biotic and abiotic stresses [10]. Given the well-established involvement of PSMs such as flavonoids, isoflavonoids, anthocyanins, terpenoids, and alkaloids in stress responses [11,12], it would be interesting to study how elicitation enhances plant defense mechanisms.

As their name suggests, elicitors (from the Latin elicere—“to draw out, to draw in”—derived from ex “out” + -licere, “to lure out, to deceive”) are substances that stimulate the biosynthesis of substances that are essential for protecting the plant as a living cellular organism during its exposure to stress. In plant tissue culture, elicitation involves the addition of elicitors at low concentrations to the growth medium under in vitro conditions to trigger a stress response and enhance the production of PSMs [13,14]. Elicitors can be classified based on their origin, site of action, and specificity of interaction with the host plant [14,15,16,17] (Table 1).

Biocompatibility, biodegradability, and non-toxicity of CTS are extensively utilized in agriculture and agrochemistry, offering substantial potential and added value for agrobiotechnology [18,19]. As a native elicitor, CTS has been extensively studied for its potential as a plant growth biostimulant, demonstrating success in enhancing root development, stimulating overall plant growth, and improving the availability and absorption of water and nutrients [20,21]. It is used to induce plant defense responses and has potential as a substitute for chemical pesticides and fungicides in agriculture [22]. Plus, CTS significantly enhances the production of PSMs, such as menthol production in *Mentha × piperita* L. [23], phenol content in *Canscora decussata* Schult [24], phenols and flavonoids in *Dracocephalum kotschyi* L. [25], lignin in *Cucumis melo* L. [26], and coumarins and furoquinolone alkaloids in common rye [27]. The low-cost production of CTS, derived from the shells of marine crustaceans, is a great example of a zero-waste economy [28].

Plant tissue culture is a fundamental technique in plant biotechnology, enabling the aseptic cultivation of plant cells, tissues, or organs in a controlled environment. Based on the principle of cellular totipotency, it allows a single plant cell to regenerate into a whole plant when provided with the appropriate nutrients and growth regulators [29]. This method is widely used to study plant development, secondary metabolite production, and stress responses in vitro. The tissue culture systems provide all the necessary nutrients, water, and energy via basal media [30], which can be customized to support the specific developmental stages of organogenesis or somatic embryogenesis. Furthermore, plant tissue culture offers a consistent platform for evaluating elicitor-induced responses, including those triggered by biotic and abiotic agents such as CTS. This makes it particularly valuable for optimizing elicitor concentration, timing, and delivery, and ideal for mechanistic studies and scalable applications in crop improvement and phytochemical production [31].

Considering the well-developed nature of the field, as evidenced by numerous comprehensive reviews [32,33,34,35,36] this review specifically explores the in vitro elicitation potential of CTS. The aim is to critically examine the methodological approaches employed and the underlying mechanisms of CTS-induced elicitation, as well as the overall effectiveness of these approaches under controlled in vitro culture conditions. Additionally, we sought to identify the existing inadequacies and knowledge gaps in the current insights into the CTS elicitation methods, with the aim of guiding future research and improving practical applications.

## 2. Chitosan (CTS): Structure and Biochemical Properties

**Figure 1:**
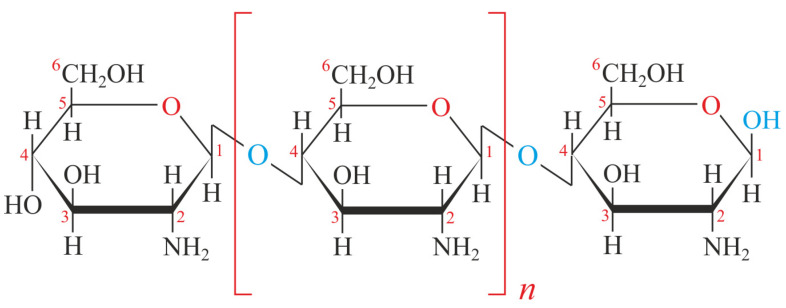
The chemical structural formula of CTS. The β-(1,4)-D-glucosamine monomer is highlighted. Originated from E. Generalic, (https://glossary.periodni.com/glossary.php?en=chitosan, Accessed on 15 August 2025).

Chitosan (CTS) is an N-deacetylated derivative of chitin, composed of β-(1→4)-linked 2-amino-2-deoxy-β-D-glucopyranose units. It is produced through the chemical conversion of chitin’s acetamide groups into primary amino groups, resulting in a polymer with significantly enhanced chemical and biochemical reactivity, primarily due to the presence of glucosamine residues [37] (Figure 1). The CTS biosynthetic pathway occurs through the coordinated action of two enzymes: chitin synthase and chitin deacetylase. Chitin synthase catalyzes the polymerization of chitin using UDP-GlcNAc (Uridine Diphosphate N-Acetylglucosamine) as a substrate, while chitin deacetylase subsequently converts chitin into CTS through enzymatic deacetylation [38,39].

Chitin, the second most abundant natural polysaccharide after cellulose, is a biodegradable mucopolysaccharide characterized by low toxicity and limited solubility in common solvents [40,41]. While it is rarely found in plant tissues, chitin is widely distributed in the exoskeletons of crustaceans and insects, as well as in the cell walls of fungi such as mushrooms. In contrast, CTS occurs naturally in shells, fish scales, mycelial cell walls of fungi and mushrooms, insects, algae, corals, and nematodes [42,43]. Crustaceans include the horseshoe crab, blue crab, Chilean crab, shrimp, prawn, lobster, and horse mussel [42]. CTS derived from fish scales and shrimp shells showed higher solubility and a higher degree of deacetylation than that of crab shells, with shrimp shells being the most suitable source, when considering additional physicochemical properties such as high molecular weight [44,45,46].

Crustacean CTS is typically obtained through a multi-step chemical process involving demineralization and deproteinization, often requiring strong acids and bases. This method yields CTS with a relatively high viscosity and molecular weight, but a lower degree of deacetylation (DD), which restricts its solubility in neutral and alkaline aqueous environments. Additionally, crustacean-derived CTS tends to contain higher levels of ash and sodium, and lower nitrogen content. A notable limitation is its potential allergenicity due to residual shellfish proteins, which may pose risks in biomedical and food-grade applications.

In contrast, fungal CTS is extracted using milder, more environmentally friendly techniques without the use of harsh chemicals. This results in a product with a significantly lower viscosity—approximately five times lower than that of crustacean CTS—and a higher DD, which enhances its solubility and chemical reactivity. Fungal CTS also exhibits lower ash content and higher concentrations of nitrogen, fiber, and fat. Importantly, it is considered hypoallergenic, making it particularly suitable for pharmaceutical, cosmetic, and food applications where biocompatibility and safety are critical [47]. Fungal species such as *Aspergillus niger*, *Trichoderma reesei*, *Rhizopus oryzae*, *Lentinus edodes*, *Pleurotus sajor-caju*, *Mucor rouxii*, *Gongronella butleri,* and *Absidia glauca* [48], along with mushroom species such as *Agaricus bisporus* [49], *Boletus bovinus* and *Laccaria laccata* [50], are some of the known sources of CTS.

The divergent biological functions of chitin and CTS can be attributed to a minor yet critical variation in the functional group at the C-2 position of the glucose residue. This structural modification is a principal factor underlying the differences in their crystalline organization, which, in turn, influences their physicochemical properties and biological activity. As a cationic polyelectrolyte, CTS possesses free amino groups on each D-glucosamine unit, enabling it to form salts upon interaction with acids. This unique property has garnered substantial global interest, positioning CTS as a versatile material for a wide range of applications across biomedical, environmental, and industrial domains [51].

The poor solubility of chitin is primarily attributed to its rigid crystalline structure, stabilized by extensive intermolecular hydrogen bonding. CTS, however, exhibits improved solubility when its amino groups are protonated, forming soluble salts in the presence of organic acids (e.g., acetic or citric acid) or mineral acids such as hydrochloric acid [51,52].

Intra- and intermacromolecular hydrogen bonding plays a critical role in stabilizing the crystalline domains of CTS in its solid state, significantly limiting its solubility in aqueous environments. Commercially available CTS, containing approximately 14 ± 2 mol% acetylated groups, is typically soluble only up to pH 6.0. However, the disruption of its crystallinity, which is achieved through chemical reacetylation, acid or enzymatic hydrolysis, or the use of physical additives such as urea and guanidine hydrochloride or ultrasonic degradation, can markedly expand its solubility options. Chemical modification (e.g., partial reacetylation) enables solubility up to pH 7.4, and physical disruption using chaotropic agents shifts the precipitation point to pH 8.0 (urea) and pH 6.5 (guanidine hydrochloride). Combined approaches allow CTS to remain soluble across a broad pH range (1–12) [53,54].

**Figure 2:**
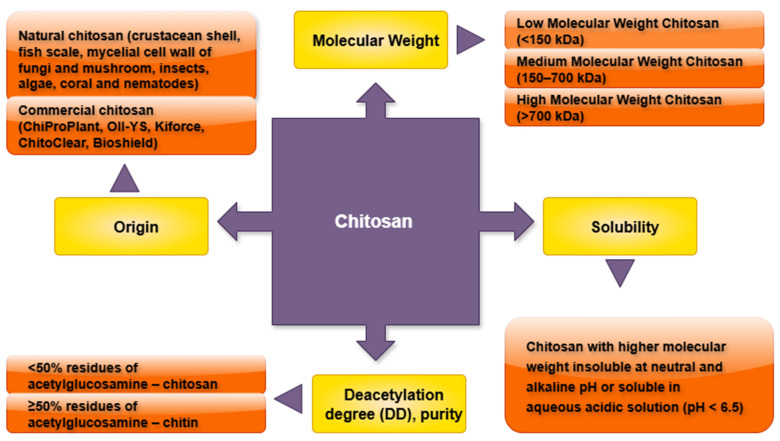
The key properties of CTS that affect its in vitro elicitation potential. DD—deacetylation degree; MW—molecular weight..

The water-soluble derivates of CTS, CTS oligosaccharides (COS), which are the primary degradation products of CTS formed through chemical or enzymatic hydrolysis, cause deacetylation and depolymerization [55]. COS have a wide range of biological applications in the induction of pathogen-related proteins [56]. Another water-soluble derivative of CTS is N-carboxymethyl CTS (CM-CTS). It is water-soluble at pH > 7, and it is used in the food and medical industries [57,58]. CM-CTS has enhanced antimicrobial activity compared to native CTS [59]. Partial reacetylation of CTS or controlled deacetylation of chitin yields a derivative known as half-acetylated CTS (HACHI), which is characterized by approximately 50% acetylation. This modification significantly enhances its aqueous solubility compared to unmodified CTS. The solubility of HACHI is further influenced by the molecular weight of the polysaccharide, with lower molecular weight variants exhibiting greater solubility [53]. Along with the above, CTS nanoparticles (CTS-NPs) are also gaining traction in the field of agriculture. In agriculture, CTS-NPs function as growth enhancers and potent antimicrobial agents against pathogenic fungi and bacteria while also serving as nanocarriers for agrochemicals, thence the term CTS-based agronanochemicals [60]. A comparison between CTS and CTS nanoparticles revealed that the inhibition zone for cariogenic *Streptococci* was significantly larger for CTS-NPs, likely due to their smaller particle size and greater affinity for bacterial cells [61]. In summary, it can be stated that the CTS efficacy and applications are largely determined by its molecular weight, degree of deacetylation, solubility, crystallinity, surface area, and particle size [62]. Figure 2 and Table 2 summarize the key properties of CTS that affect its use.

There are several commercially available CTS products, such as Chito Plant™ by ChiPro GmbH, OII-YS™ by Venture Innovations, Kiforce™ by Alba Milagro, ChitoClear™ by Primex ehf, and Bioshield™ by Seafresh [63]. Worldwide, there are many companies focused on innovation in the extraction techniques, product formulations, and sustainability practices to meet the growing demand in the pharmaceutical and cosmetic industry, as well as in agriculture. They offer a wide range of chitin-based, mushroom-based, and crustacean-based CTS products, products that include CTS derivatives, treated CTSs, and synthesized CTSs. They customize the options for specific research or industrial needs. Individual CTS-based products differ in the molecular weight, the degree of deacetylation (DDA%), viscosity, and purity. The list of some CTS supply companies is provided in Table 3.

## 3. Molecular Mechanisms of CTS Action

### 3.1. CTS Effects on the Induction of Defence Genes

CTS plays a key role in activating the plant defense systems by triggering the expression of genes involved in both local and systemic responses. It boosts the production of phenolic compounds and carbohydrates, which are important for strengthening plant immunity. CTS also enhances crop resistance to various abiotic stresses by modulating the signaling pathways—such as those related to lipid peroxidation—and regulating the expression of specific stress-responsive genes (Table 4). These include the genes encoding antioxidant enzymes, AP2-domain transcription factors responsive to octadecanoid derivatives, mitogen-activated protein kinases (MAPKs), and geissoschizine synthase, all of which contribute to improved stress tolerance and overall plant resilience [20]. CTS induces systemic acquired resistance by stimulating the transcription of phenylalanine ammonia-lyase, defense system-associated protein-1, and peroxidase (POX) [64]. Under salinity stress, CTS upregulates the expression of the *AsHKT1*, *AsNHX4*, *AsNHX5*, and *AsNHX6* genes, which encode the Na^+^/H^+^ exchangers in *Agrostis stolonidera* L. (creeping bentgrass) [65]. CTS improved the tolerance of *Ocimum basilicum* L. (sweet basil) to salinity by influencing the phenylalanine ammonia lyase (PAL) and chavicol O-methyltransferase (CVOMT) genes involved in the phenylpropanoid pathway, leading to an increase in phenolic compounds [66]. CTS treatment significantly enhanced the expression of *MAPK3*, *GS,* and *ORCA3* genes under salinity stress [67]. In *Solanum lycopersicum* L., CTS leads to the overexpression of the superoxide dismutase (*SOD*) and jasmonic acid (*JA*) genes under salt stress [68] and the *HsfA1a*, *SlAREB1*, *LeNCED1,* and *LePIP1* genes under drought stress [69]. The application of CTS to stressed *Catharanthus roseus* (L.) plants significantly enhanced the expression of *STR, DAT, PRX1*, and *GS* genes under drought conditions [70]. CTS enhances the expression of cold tolerance-related genes such as *Chit134*, *BSK2*, *ERF*, *NCED,* and *DRE326* in *Kobresia pygmaea* (Willd.) [71]. CTS can increase heat tolerance by inducing the abscisic acid (ABA) activity and further inducing the ABA-responsive genes related to plant defense [72].

### 3.2. Physiological and Biochemical Effects of CTS on Plant Cellular Functions

CTS has been reported to increase photosynthesis by stimulating the enzymes involved in nitrogen and carbon metabolism. It also affects the dark and light responses of the photosynthetic apparatus [56]. CTS intervenes in many physiological events in the plant cells, such as the antioxidant activity of the reactive oxygen species (ROS), superoxide anion and free hydroxyl radicals [73], and hydrogen peroxide (H_2_O_2_) accumulation [74]. CTS can induce the synthesis of H_2_O_2_ in plant cells, thereby triggering defense responses against biotic and abiotic stress factors while also enhancing the activity of key antioxidant enzymes, such as POX, SOD, and catalase (CAT), which are involved in the direct neutralization of ROS [75]. CTS was applied at a concentration of 1% following salt stress in Catharanthus roseus L., delaying the reduction of chlorophyll and inducing the POX, CAT, glutathione, ascorbate, and reductase activities [67]. Applying 0.5 mg/L of CTS to the *Silybum marianum* L. suspension cell culture medium resulted in a high antioxidant activity [76]. Using the CTS in nettle (*Urtica dioica* L.) showed a significant increase in the antioxidant enzyme activities, such as polyphenol oxidase, POX, and phenylalanine ammonia-lyase [77]. Treating the *Trifolium repens* L. seeds with 5 mg/L of CTS under water stress increased the accumulation of ROS and provided better control of membrane lipid peroxidation during the seed germination [78].

CTS slows down cell aging within the host plant cell membrane, preventing mass loss. It stabilizes the humidity and acidity levels by delaying the dissolution of soluble sugars and other solids within the cell [79]. CTS promotes the expression of enzymes in the phenylpropanoid pathway, such as PAL, and activates the hormone-mediated signaling cascades (e.g., ABA), which may influence membrane transport and cellular homeostasis [80]. It can inhibit the H^+^-ATPase activity in the cell membrane, increase the activation of MAP kinases, and increase the concentration of Ca^2+^ ions [81]. At the same time, it can penetrate into the cell cytoplasm and change the permeability of the cell membrane [82]. It has also been shown that when applied to seeds and seedlings, CTS can increase plant nutrient absorption, chlorophyll content, and the rate of photosynthesis [83]. In *Lilium regale* (Wils.), it was revealed that the highest and lowest chlorophyll content was obtained in CTS at 200 ppm and 0 ppm (control), respectively [84]. CTS treatment enhanced the stomatal aperture and expanded the pore size under varying levels of drought stress in sugar beet plants [85]. CTS reduces the stomatal conductance and enhances leaf resistance to water vapor loss in maize under drought conditions [86]. The application of CTS stimulates the stomatal closure through ABA synthesis under drought stress in *Phaseolus vulgaris* L. [87]. 

## 4. CTS’s Potential Under In Vitro Conditions

### 4.1. Methods of Plant Tissue Culture: Techniques, Applications, and Advantages

The plant tissue cultures serve as powerful tools for plant propagation, genetic engineering, and metabolite production under sterile conditions. These methods are foundational to plant biotechnology. Their versatility and efficiency continue to drive innovation in agriculture, industry, and biotechnology.

Among these, micropropagation is widely employed for the rapid clonal propagation of genetically uniform and disease-free plants, making it ideal for commercial horticulture and conservation of rare species [88]. It uses small explants, typically meristematic tissues, and proceeds through stages including initiation, multiplication, rooting, and acclimatization. The optimization of culture media and plant growth regulators remains critical, with cytokinins and auxins playing pivotal roles in shoot and root induction, and emerging evidence suggests that low-dose stressors may stimulate proliferation [89].

The callus culture involves the induction of undifferentiated cell masses (calli) from explants on solid media enriched with plant growth regulators. These calli can be used for regeneration or genetic modification [90,91]. Recent studies show the diversity of callus types—ranging from friable and compact to embryogenic and rhizogenic—each with distinct morphological and physiological traits that influence their utility in vitro [92]. The induction and maintenance of callus cultures are highly dependent on the balance of plant growth regulators, particularly auxins and cytokinins, which modulate cellular dedifferentiation and proliferation. Advances in multi-omics approaches have revealed the species-specific hormone profiles in callus tissues, suggesting new avenues for metabolic engineering [93]. Moreover, callus cultures serve as precursors for cell suspension systems, which are increasingly used in a scalable production of bioactive compounds.

Plant cell cultures have become increasingly important as biofactories for the production of high-value metabolites, recombinant proteins, and therapeutic compounds. Recent research emphasizes the optimization of cell suspension cultures, which are derived from friable callus and maintained in liquid media under controlled conditions. These cultures offer scalability and reproducibility, making them ideal for industrial applications [94,95].

Hairy root cultures have become a powerful biotechnological platform for the sustainable production of high-value secondary metabolites in plants [96]. Recent studies emphasize their utility in producing alkaloids, flavonoids, terpenes, and other bioactive compounds with pharmaceutical relevance, particularly in medicinal plant species. Advances in metabolic engineering and elicitation strategies have further enhanced metabolite yields [97].

### 4.2. CTS Application in Plant Tissue Cultures

CTS is used to mimic environmental stress under in vitro conditions and stimulate the production of PSMs. The elicitor-mediated in vitro synthesis ensures a consistent production of PSMs while providing a higher standard of quality and product uniformity [24]. When used as an elicitor, CTS initiates the increased synthesis of phenolic compounds and carbohydrates [98]. CTS has been widely recognized for its ability to stimulate the synthesis of phytoalexin proteins, which play a pivotal role in plant defense mechanisms [74]. These proteins not only contribute to direct antimicrobial activity but also serve as key modulators of fundamental signaling pathways, including those governed by ethylene, salicylic acid, and jasmonic acid [99]. Enzymatic and metabolic analyses proved that the mixture of CTS nanoparticles and methyl jasmonate improved and prolonged the activity of phenylalanine ammonia-lyase enzyme and the production of phenols and flavonoids in suspension cell cultures [100]. Using CTS to stimulate the callus medium of a *Ginkgo biloba* L. tree, increased the content of flavonoids to 2.55 mg/g DW using the dose of CTS (50 mg/L), while the content of flavonoids in the control callus medium was 1.57 mg/g DW [101]. The effects of CTS on the production of PSMs in *Iberis amara* L. cell suspension cultures were investigated, revealing that a 50 mg/L dose of CTS led to an approximately 2.19-fold increase in flavonoid content compared to the control callus [102]. According to the variance analysis results in *Lilium regale* Wils., the flavonoid content at different CTS concentrations was found to be significant at a 1% probability level [84]. The combination of CTS and methyl jasmonate decreased the antioxidant capacity in broccoli sprouts, and this mixture decreased the total polyphenols content compared to the control sprouts [103]. The induction of hairy roots in *Scutellaria bornmuelleri* L., an important medicinal plant, was studied by applying methyl jasmonate, methyl-b-cyclodextrin, and CTS alone or in combination for induction. The mixture of methyl jasmonate and CTS increased the production of wogonin and baicalein by 9.15 and 10.56, respectively [104]. The *Hyptis suaveolens* JACQ. roots secrete podophyllotoxin, a lignan that has an important role in the pharmaceutical industry. CTS supplementation resulted in a 2.1-fold increase in the podophyllotoxin synthesis from the root cultures in a liquid medium compared to the control [105]. A positive effect of CTS in the culture medium was observed, where it prevented browning of the culture while stimulating root growth [106]. All CTS concentrations (0.0, 10.0, 20.0, and 30.0 mg/L) promoted the regeneration of *Melissa officinalis* L. callus cultures, although the highest regeneration was obtained at a 30.0 mg/L CTS concentration [107].

**Figure 3:**
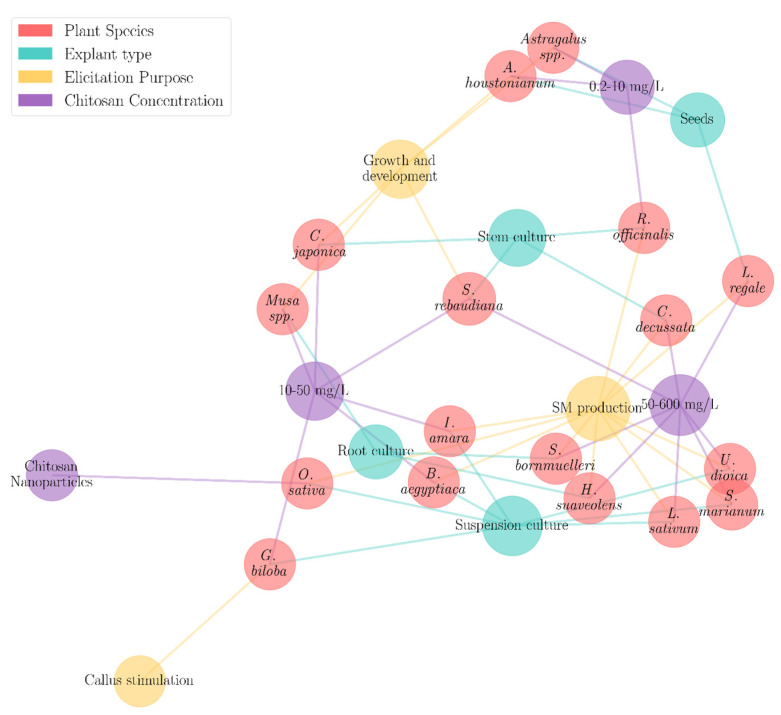
CTS in vitro applications in different plant species. This figure provides a graphical representation of the inter-relationship between plant species and other parameters such as explant type, elicitation purpose, and CTS concentration. Different colours are used to represent the nodes related to these parameters. The red colour denotes the plant species. The green, blue, and violet colours represent explant Type, elicitation purpose, and CTS concentration, respectively. The Fruchterman–Reingold model [117] was used to draw the graph.

Table 5 summarizes the CTS in vitro elicitation applications. The data have been structured in a tabular format for clarity (Table 5), and a network model was constructed to facilitate the visualization of pre-existing relationships (Figure 3). The graph follows the spring layout, which is built on the inference of the Fruchterman–Reingold model [108], a widely used force-directed algorithm for graph drawing. Through this process, clusters and communities can be clearly visualized as tightly connected nodes, which are brought closer, and less connected ones, which are pushed apart [108]. Further, an initiative has been taken to increase the size of the nodes based on the connection. The nodes with a higher number of connections, i.e., of bigger sizes, are enclosed within a cluster. The nodes with fewer connections are mostly placed at the outer perimeter of the graph. This graph primarily studies the relationship of plant species with other parameters. Hence, every plant species node has exactly three edges unless there are multiple associated parameters of the same type, whereas other nodes can have varying edges, starting with one. Similarly, a CTS concentration of 50–600 mg/L shows eight connections producing a prominent cluster. Notably, a significant number of nodes of plant species have common connections of a CTS concentration of 50–600 mg/L and callus stimulation, which can be visible in Figure 3 as a significant number of plant species nodes (shown within a tight circle).

## 5. Inadequacies in Current Insights into CTS Elicitation Methods

The in vitro plant cultures utilize nutrient-rich media and offer a controlled, aseptic environment for the growth of plant cells, tissues, and organs, leading to an enhanced production of plant secondary metabolites compared to in vivo plants [118]. The different types of in vitro plant cultures include the callus culture, cell suspension culture, organ culture, protoplast culture, stem culture, and hairy root culture [119,120]. Given the diversity of biochemical and biological properties of CTS, one of the aims of this study was to summarize the common features of the CTS elicitation methods in in vitro plant cultures. As mentioned above, several review studies have competently elaborated on the structure and chemical properties of CTS [62,121,122]. However, when searching for information on the elicitation potential of CTS for the in vitro condition, we encountered incomplete information on the origin, type, degree of purity, and deacetylation of CTS, as well as the method of application of CTS-based solutions for in vitro elicitation. CTS exhibits high thermal stability, and it degrades only at temperatures above 280 °C [62], whereas autoclaving is typically performed at 120 °C. This suggests that CTS can be added before autoclaving; however, it remains unclear whether its stability at this temperature is affected by variations in the type or the properties of the CTS used. The literature does not indicate whether the reported thermostability at the autoclave temperature is influenced by the type and properties of the CTS used. The information on the methodological approach is provided in Table 6, more or less in its original version. The formal changes concern the units of measurement (e.g., g/L or mg/L), replacement of abbreviations by full words, and minor stylistic adjustments.

The most common missing information includes (Table 6):

- *CTS type based on the molecular weight, degree of deacetylation, and purity:* Variations in the molecular weight, degree of deacetylation, and purity of CTS may lead to differential responses in the plant tissue cultures. These physicochemical properties are likely to influence its role as a growth enhancer or elicitor of secondary metabolites.
- *Method of preparing the CTS stock solutions and the subsequent sterilization approach:* The procedure used to dissolve and sterilize CTS may alter its structural integrity and effectiveness. Inappropriate preparation methods could reduce its bioavailability or biological activity in cultured tissues.
- Method of adding the required CTS concentration to the medium: The timing and method of incorporating CTS into the culture medium may impact its uniformity and interaction with plant cells. This could influence both the growth outcomes and secondary metabolite production.

**Figure 4:**
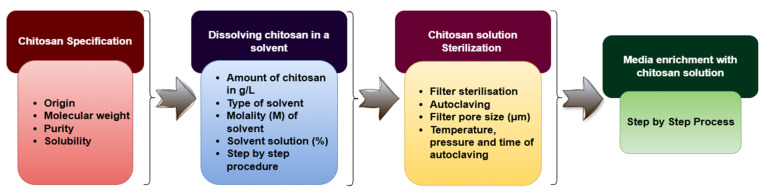
Required steps for in vitro CTS elicitation method.

In order to accurately and efficiently exploit the elicitation potential of the native CTS polymer, it is necessary to specify the individual steps in the preparation of this elicitation process (Figure 4).

## 6. Future Perspectives and Concluding Remarks

CTS exerts a lethal effect on a wide range of organisms, including fungi, bacteria, viruses, nematodes and insects, effectively contributing to the control and suppression of these pathogens and pests [126,127,128,129]. It has the ability to stimulate the production of phytoalexins, proteinase inhibitors, pathogenesis-related proteins, and plant defense enzymes in response to biotic stress [130]. CTS can be used as a pesticide either on its own or in combination with other products to combat plant diseases caused by bacteria, fungi, and other pests and insects while also aiding in seed coating, promoting plant growth, and supporting postharvest [18].

Rapid climate change is making the world’s agriculture vulnerable to various abiotic stresses, which decrease crop yields by 51–82% [131], which, in turn, seriously threatens food security. It has already been established that CTS can be beneficial in the amelioration of abiotic stress by increasing the crop yield, as reviewed in [72]. The co-application of CTS and silicon improved grain yield in drought-affected wheat [132]. The application of CTS improved the plant height, leaf count (tiller), fresh and dry weight of tiller, root length, and photosynthetic pigments in the salinity-treated maize plants [133,134]. A foliar application of CTS in tomato improved the plant height, leaf count, leaf area, stem diameter, fruit count, fruit firmness, chlorophyll content, and yield [135]. The application of CTS in common bean plants increased the cellular concentration of antioxidant system components and proline, resulting in reduced oxidative stress, membrane damage, and electrolyte leakage, and ultimately improved plant yield [136]. When wheat was grown in the soil mixed with wastewater contaminated by Pb, Cd, Cr, Ni, Cu and Co, a soil application of CTS effectively reduced the bioavailability of the HMs, decreased HMs concentration in roots, shoots, and grains up to 89%, and improved the photosynthesis, plant growth, grain yield, and nutrition in the HM-stressed plants [137]. Another study reported that a foliar application of 750 ppm of CTS on cauliflower plants improved the vegetative growth parameters, yield, physical quality of the head, leaf mineral content (N, P and K), leaf relative water content, membrane stability index, total chlorophyll and nutritional values (vitamin C and crude protein) under heat stress [138]. CTS and its nanoparticles enhance the plant tolerance to drought stress by boosting the plant growth, photosynthetic pigments, endogenous indole acetic acid, activities of antioxidant enzymes, while reducing the levels of hydrogen peroxide (H_2_O_2_) and malondialdehyde [139]. It enhances plant growth and mitigates the effects of salinity stress by regulating the cellular osmotic pressure by improving water availability, which increases the uptake of essential nutrients [140]. CTS has been shown to enhance cold tolerance by affecting photosynthesis, antioxidant enzyme activities, transcriptomic responses, and phenylalanine metabolism [71].

## 7. Conclusions

The aim of this review was to collect the available knowledge in the area of the in vitro application potential of CTS elicitation and its methodology. The results of the review study indicate that methodological procedures for the preparation of CTS elicitation solutions and their application to culture media are often absent or incomplete. In a number of cases, the origin and the type of CTS are not specified. Further research should aim to deepen our understanding of the molecular mechanisms underlying the CTS activity and explore its integration into the existing plant tissue cultivation practices, taking full advantage of its beneficial properties. As the efficacy of CTS is dependent on the plant species, CTS concentration, and molecular weight, the applications of CTS should be properly studied. Finally, bridging the gap between the controlled in vitro studies and variable field conditions, alongside the standardization of CTS preparation and application methods, is essential to fully realize the potential of CTS in sustainable and effective agricultural practices.

## Figures and Tables

**Table 1:** Classification of elicitors.

Factor	Type of Elicitor	Examples
Site of action	Exogenous elicitors	Molecules produced by pathogens that act externally on the plant.
	Endogenous elicitors	Compounds synthesized by the plant itself in response to pathogen attack.
Origin	Biotic elicitors	Derived from living organisms or their metabolic products: - polysaccharides (chitin, CTS, glucans, etc.); - oligosaccharides (mannuronate, galacturonides, etc.); - proteins (oligandrin, glycoproteins, etc.).
		Biotic elicitors with a defined composition: CTS, alginate, pectin, chitin, elicitin, and pectic fragments.
		Biotic elicitors with a complex composition: fungi homogenate, yeast extract, and fungal spores.
	Abiotic elicitors	Originating from non-living sources.
		Chemical elicitors: silicon, benzothiadiazole, ethanol, acetic acid, and metal ions (e.g., Cu^2+^, Zn^2+^, Ag^+^).
		Physical elicitors: UV radiation, temperature stress, drought, osmotic and saline stress, and mechanical damage.
Specificity	General elicitors	Capable of inducing defense responses in a wide range of plant species.
	Race-specific elicitors	Trigger resistance only in plants carrying specific resistance genes.

**Table 2:** CTS properties (modified from Román-Doval et al. (2023) [62]).

Properties	Characteristic	
Molecular weight (MW)	High (HMWC)	>700 kDa
	Medium (MMWC)	150–700 kDa
	Low (LMWC)	<150 kDa
	100,000–1,200,000 Da, industrial CTS.	>1,000,000 Da, native chitin.
Deacetylation degree (DD)	100%, the biopolymer contains only monomeric forms of 2-amido-2-deoxy-D-glucopyranose.	50%, the biopolymer contains 50% of 2-amido-2-deoxy-D-glucopyranose units.
	100% deacetylation, CTS.	0% deacetylation, chitin.
Residues of acetylglucosamine (2-acetamido-2-deoxy-D-glycopyranose)	<50%, the substance is categorized as CTS.	≥50%, the substance is categorized as chitin.
Solubility	100% DD, soluble in water.	50% DD, soluble in an aqueous acidic environment.
	HMWC; lower solubility in water.	LMWC; higher solubility in water.
Crystallinity index (CI)	CTS swelling, its porosity, water absorption, and moisture retention are affected by crystallinity.	Both, chitin (0% deacetylated) and CTS (100% deacetylated) have the highest crystallinity value. The solid form of CTS is semi-crystalline.
Particle size	<1 mm, often used in most applications.	
Surface area	<10 m^2^/g, CTS flakes or powder.	
High temperature sensitivity	>280 °C; heat degradation of CTS.	
Antioxidant activity	LMWC; higher.	HMWC; lower.
Bioactivity (in terms of biocompatibility, nontoxicity, biodegradability, antimicrobial activity, etc.)	LMWC; more significant.	HMWC; less significant.

Notes: LMWC—low-molecular-weight CTS; MMWC—medium-molecular-weight CTS; HMWC—high-molecular-weight CTS; DD—deacetylation degree.

**Table 3:** This table presents some companies that supply CTS.

Company Name	Country	Focus Areas
Advanced Biopolymers AS	Norway	Industrial and medical-grade CTS
Axio Biosolutions	USA	CTS-based wound care products
BIO21 Co. Ltd.	Thailand	CTS for agriculture and water treatment
ChytoLytic	Canada	Pure, clean, high-quality, high-grade CTS for advanced R&D and commercial biomedical, pharma, and industrial applications
Golden-Shell Pharmaceutical	China	Pharmaceutical-grade CTS
Heppe Medical CTS GmbH	Germany	High-purity CTS for biomedical use
Kimica Corporation	Japan	Industrial and cosmetic applications
KitoZyme S.A.	Belgium	Biopolymers for healthcare and nutrition
Meron Biopolymers	Norway	Eco-friendly biopolymers for food and cosmetics
Panvo Organics Pvt. Ltd.	India	Sustainable CTS for agriculture, pharma

**Table 4:** Summary of CTS-mediated gene expression and physiological responses in various plant species under abiotic stress conditions.

Plant Species	Stress Condition	Genes Affected	Biological Function/Pathway	Reference
Various species	General defense response	*PAL*, *PR1*, *POX*	Induces systemic acquired resistance (SAR); enhances phenolic and carbohydrate production.	[54]
*Agrostis stolonifera* L. (creeping bentgrass)	Salinity stress	*AsHKT1*, *AsNHX4*, *AsNHX5*, *AsNHX6*	Regulates Na^+^/H^+^ exchangers; improves ion homeostasis.	[55]
*Ocimum basilicum* L. (sweet basil)	Salinity stress	*PAL*, *CVOMT*	Activates phenylpropanoid pathway; increases phenolic compound synthesis.	[56]
Various species	Salinity stress	*MAPK3*, *GS*, *ORCA3*	Enhances stress signaling and secondary metabolite biosynthesis.	[57]
*Solanum lycopersicum* L. (tomato)	Salt stress	*SOD*, *JA*	Boosts antioxidant defense and jasmonic acid signaling.	[58]
*Solanum lycopersicum* L. (tomato)	Drought stress	*HsfA1a, SlAREB1, LeNCED1, LePIP1*	Improves drought tolerance via ABA signaling and aquaporin regulation	[59]
*Catharanthus roseus* L.	Drought stress	*STR*, *DAT*, *PRX1*, *GS*	Enhances secondary metabolite production and oxidative stress defense.	[60]
*Kobresia pygmaea* (Willd.)	Cold stress	*Chit134*, *BSK2*, *ERF*, *NCED*, *DRE326*	Activates cold-responsive transcription factors and ABA-related genes.	[61]
Various species	Heat stress	*ABA-responsive genes*	Increases heat tolerance via ABA signaling and defense gene activation.	[62]

The section above Table 4 explains the gene abbreviations.

**Table 5:** CTS in vitro elicitation applications.

Species	Types of Tissue/Explants Used in the Culture Media	Purpose	CTS Concentration (mg/L)/Form	Reference
*Ageratum houstonianum* Mill.	Seeds	In vitro seed germination and organ development	2.5, 5.0, 10.0 mg/L, (10%, 20% DA shrimp CTS)	[109]
*Astragalus spp.*	In vitro seedlings	Growth stimulation	0.2, 0.5, 1, 2, 3, and 4 mg/L, (CTS nanoparticles)	[110]
*Balanites aegyptiaca* L.	Callus suspension culture	SM production	40 mg/L	[111]
*Canscora decussata* Schult.	Nodal explants	SM production	200 mg/L (Sigma-Aldrich)	[24]
*Citrus japonica* THUMB.	Nodule stems	Shoot multiplication	10, 15, 20, 25 mg/L	[112]
*Ginkgo biloba* L.	Callus suspension culture	Callus stimulation	50 mg/L	[101]
*Hyptis suaveolens*JACQ.	Root culture	Podophyllotoxin synthesis	50; 100; 150 mg/L (≥98% purity, Sigma-Aldrich)	[105]
*Iberis amara* L.	Cell suspension culture	SM production	50 mg/L	[102]
*Lepidium sativum* L.	Callus suspension culture	SM production and antioxidant activity	100, 250 and 500 mg/L (low molecular weight, 50,000 Da)	[113]
*Lilium regale*Wils.	In vitro seedlings	Flavonoid content, chlorophyll, and regeneration	50; 100; 150; 200 mg/L	[84]
*Musa spp.*	Rhizome and sucker	Regeneration of shoots and roots	25 mg/L(shrimp CTS)	[114]
*Oryza sativa* L. *japonica*	Cell suspension cultures	SM production	CTS nanoparticles (shrimp CTS, ≥75% DA)	[100]
*Rosmarinus officinalis* L.	Apex and lateral buds	Callus biomass, SM production	5 mg/L	[115]
*Scutellaria bornmuelleri* L.	Hairy root cultures	Flavonoid content	50,100, 200 mg/L	[104]
*Silybum marianum* L.	Cell suspension cultures	Silybin production	600 mg/L	[116]
*Stevia rebaudiana* L.	Nodal stems	Shoot regeneration	20, 40, 60, 80, 100 mg/L (low, medium, high MW CTS)	[117]
*Urtica dioica* L.	Callus suspension culture	SM production	50 and 100 mg/L	[77]

DA—deacetylation, SM—secondary metabolites.

**Table 6:** Methodological shortcomings of CTS elicitation in vitro.

Methodology of CTS Elicitation In Vitro	Missing Information	Reference
The stock solution of 0.3 g/L CTS (≥ 98% purity; Sigma-Aldrich) was prepared by dissolution in 1000 mL of distilled water, to which 10 mL of acetic acid was added. From this stock solution, solutions with concentrations of 50.0, 100.0, and 150.0 mg/L were prepared.	CTS stock solution sterilization Medium supplementation with CTS	[105]
CTS was dissolved in 5% (*v*/*v*) 1 N HCl through gentle heating and continuous stirring and added to the callus induction medium at concentrations of 200, 400, and 800 mg/L.	CTS type CTS stock solution sterilization Medium supplementation with CTS	[123]
Soluble CTS (ChitoPlant, ChiPro GmbH Bremen, CTS content 99.9%) was added to the rooting medium prior to autoclaving, and the pH was adjusted to 5.7–5.8. The concentrations of soluble CTS of 0, 5, 15, 50, 150, 500, 750, and 1000 mg/L were tested.	CTS solutions preparation	[124]
CTS was added in six different combinations into the MS medium prior to the adjustment of pH and medium autoclaving.	CTS type CTS solutions preparation	[115]
MS medium containing different molecular weights (low, medium, and high) and concentration (0, 20, 40, 60,80, and 100 mg/L) of CTS.	CTS type CTS solutions preparation and sterilization Medium supplementation with CTS	[117]
CTS (Sigma-Aldrich) was added on the 8th day into the medium (200 mg/L).	CTS solutions preparation and sterilization Medium supplementation with CTS	[24]
CTS at a concentration of 10, 15, 20, and 25 mg/L.	CTS type CTS solutions preparation and sterilization Medium supplementation with CTS	[112]
Commercially purchased Sigma-Aldrich CTS solutions were dissolved in distilled warm water and sterile filtered through a prefilter (0.2 m pore size; Advantec). The sterilized CTS solutions at a concentration of 20, 40, and 80 mg/L were added to the callus culture flasks.	CTS type	[111]
Low-molecular-weight CTS (50,000 Da) (Sigma-Aldrich, Taufkirchen, Germany) was used in this study.	Complete information	[113]
Low-molecular-weight CTS (50,000 Da) (Sigma-Aldrich, Germany) was dissolved in 3% (*v*/*v*) 0.1 M acetic acid using gentle heating and continuous stirring (at 60 °C, 12 h with stirring). The pH was adjusted to 5.8 with 1 N sodium hydroxide (NaOH), and the final concentration was adjusted to 10 mg×mL^−1^. The solution was stirred to further dissolve the CTS and then autoclaved for 15 min at 121 °C. The solution was kept at 4 °C prior to use. The CTS was added to the sub-cultures at two final concentrations (25 and 50 mg/L).	Complete information	[125]
